# *ESR1* Gene Mutations and Liquid Biopsy in ER-Positive Breast Cancers: A Small Step Forward, a Giant Leap for Personalization of Endocrine Therapy?

**DOI:** 10.3390/cancers15215169

**Published:** 2023-10-27

**Authors:** Margaux Betz, Vincent Massard, Pauline Gilson, Andréa Witz, Julie Dardare, Alexandre Harlé, Jean-Louis Merlin

**Affiliations:** 1 Département de Biopathologie, Institut de Cancérologie de Lorraine, CNRS UMR 7039 CRAN, Université de Lorraine, 54519 Vandœuvre-lès-Nancy, France; 2 Département d’Oncologie Médicale, Institut de Cancérologie de Lorraine, 54519 Vandœuvre-lès-Nancy, France; v.massard@nancy.unicancer.fr

**Keywords:** endocrine therapy, breast cancer, liquid biopsy, *ESR1* gene

## Abstract

**Simple Summary:**

Endocrine therapy (ET) remains the mainstay of treatment for Hormone Receptor-positive breast cancer, both in the early and advanced settings. The acquisition of mutations in the *ESR1* gene encoding the estrogen receptor represents one of the main resistance mechanisms to ET. A conventional tumor tissue biopsy can be used to detect *ESR1* mutations; however, these mutations are less likely to present on initial tissue biopsy, and such an invasive approach is hardly repeatable over time for longitudinal disease monitoring. The research of *ESR1* mutations in liquid biopsies based on the analysis of circulating cell-free DNA in plasma is of increasing interest and has been recently recommended to guide therapy in patients with estrogen receptor-positive breast cancers at progression under ET. This comprehensive review reports the most recent advances and recommendations in this field and compares the different techniques available for the analysis of the *ESR1* gene in liquid biopsy.

**Abstract:**

The predominant forms of breast cancer (BC) are hormone receptor-positive (HR+) tumors characterized by the expression of estrogen receptors (ERs) and/or progesterone receptors (PRs). Patients with HR+ tumors can benefit from endocrine therapy (ET). Three types of ET are approved for the treatment of HR+ BCs and include selective ER modulators, aromatase inhibitors, and selective ER downregulators. ET is the mainstay of adjuvant treatment in the early setting and the backbone of the first-line treatment in an advanced setting; however, the emergence of acquired resistance can lead to cancer recurrence or progression. The mechanisms of ET resistance are often related to the occurrence of mutations in the *ESR1* gene, which encodes the ER-alpha protein. As *ESR1* mutations are hardly detectable at diagnosis but are present in 30% to 40% of advanced BC (ABC) after treatment, the timeline of testing is crucial. To manage this resistance, *ESR1* testing has recently been recommended; in ER+ HER2− ABC and circulating cell-free DNA, so-called liquid biopsy appears to be the most convenient way to detect the emergence of *ESR1* mutations. Technically, several options exist, including Next Generation Sequencing and ultra-sensitive PCR-based techniques. In this context, personalization of ET through the surveillance of *ESR1* mutations in the plasma of HR+ BC patients throughout the disease course represents an innovative way to improve the standard of care.

## 1. Introduction

The predominant forms of breast cancer (BC) are hormone receptor-positive (HR+) HER2 tumors expressing estrogen (ER+)—and usually progesterone—receptors (PR+). HR+ BCs can benefit from endocrine therapy (ET), which targets either the estrogen receptor (ER) or its ligand, 17b-estradiol, the main form of estrogen. Three types of ET are approved as a treatment for ER+ BCs. Historically, selective ER modulators (SERMs), such as Tamoxifen, have been developed, followed by aromatase inhibitors (AIs), such as Exemestane and Letrozole, which block the conversion of androgens to estrogens, and more recently, selective ER downregulators (SERDs) such as Fulvestrant [1]. ET is the mainstay of adjuvant treatment in the early setting and the backbone of first-line treatment in an advanced setting, alone or combined with (2) cyclin-dependent kinase (CDK)4/6 inhibitors.

Patients with early BC still have a substantial risk of recurrence despite adjuvant ET, and the majority of patients with ABC undergoing first-line ET combined with CDK4/6 inhibitors experience disease progression within 5 years (3,4). Various mechanisms of resistance have been studied, including the emergence of mutations in the ESR1 gene, which encodes the ER-alpha (ERα) protein (3,5,6).

To prevent or manage this resistance, numerous treatment options have been investigated. The testing of *ESR1* mutations has recently become of great interest in ER+ HER2− ABCs, leading the latest American Society of Clinical Oncology (ASCO) Guideline Rapid Recommendation Update [2]. In recent years, circulating tumor DNA (ctDNA) has proven to be a more accurate, reliable, and convenient way to capture *ESR1* mutations, making liquid biopsy a great option for regular testing using either Next Generation Sequencing (NGS) or digital droplet PCR (ddPCR). Taken altogether, this creates a new guideline in terms of mutation monitoring for ABC patients, based on the surveillance of *ESR1* mutations throughout the disease and the proposal of treatment adjustments upon the rise of *ESR1* mutations.

## 2. The Road to Resistance to Endocrine Therapy: The Role of *ESR1* Gene

### 2.1. Estrogen Receptor: Signaling Pathway and ESR1 Gene

The estrogen receptors are involved in many physiological cellular processes, such as cell proliferation, survival, and differentiation. Their activation depends on the binding of 17β-estradiol, the primary form of estrogen in humans. ERα, ER-beta (ERβ), and G-protein-couple estrogen receptor 1 (GPER1) are the three predominant estrogen receptors; ERα subtype accounts for one of the most studied subtypes and is encoded by the *ESR1* gene. The nuclear protein acts as a ligand-dependent transcription factor, modulated by two activation domains, AF-1 and AF-2, the latter being the only domain allowing the activation of ERα and resulting in genomic activity [3]. The binding of estrogens to ERs forms the estrogen receptor–dimer complex, which translocates to the nucleus and recognizes the estrogen response element (ERE).

The ligand binding domain (LBD) is composed of twelve α-helices sandwiched between two β-sheets [4]. Structurally, the twelfth helix (H12) of the LBD plays a key role in the conformation modification of the receptor. The agonist conformation, also known as “apo” conformation, occurs after the binding of estrogen and allows the recruitment of coactivators provided by the proper folding of H12. On the contrary, antagonists interfere with H12 folding and prevent the recruitment of coactivators, thus blocking ERα activity [5,6].

Besides its role as a transcription regulator within the nucleus, a part of the ERα activity is nongenomic. ERα can be bound to the plasma membrane and interacts with signal transducing factors following estrogen binding. The estrogen receptor homodimer functions with signaling molecules such as Phosphoinositide 3-kinase (PI3K) [7], which in turn signals to AKT (protein kinase B), activating the PI3K/ATK/mTOR (mammalian target of rapamycin) pathway [8]. It is involved in cell proliferation, survival, invasion, and other major cell functions [9]. ER signaling also promotes cell cycle progression through the binding to the *CCND1* promoter, coding for the cyclin D, and modulating the mitosis process [10]. This cyclin binds to the CDK4/6 complex, governing the mid-G1 phase of the cell cycle [11]. Taken altogether, this shows that ERα is involved in essential signaling pathways, which can be regulated by the integrity of the *ESR1* gene and its LBD.

### 2.2. Endocrine Therapy

There are currently three classes of therapies approved for ER+ BCs targeting ERα directly or indirectly: SERMs, SERDs, and AIs. Other pathways can be targeted simultaneously through PI3K inhibitors, mTOR inhibitors, or CDK4/6 inhibitors, for example.

#### 2.2.1. Selective Estrogen Receptor Modulators: SERMs

SERMs were designed to act as antiestrogen and compete with the ligand of ERα in order to prevent the correct folding of H12, thus blocking coactivator binding and ERα activation [12]. SERMs can be divided into separate categories based on their chemical structures; Triphenylethylenes, also known as Tamoxifen (TAM) or “tamoxifen-like”, account for the most used SERMs in clinical practice [13]. They have an agonist or antagonist action depending on the type of tissue targeted.

Originally developed as a contraceptive in the 1950s, TAM’s applications were later orientated toward cancer research and made it the first drug used both for the treatment and prevention of BC [14]. Biologically, its anticancer antagonistic properties come from the hydroxylation of TAM by the isozymes CYP2D6 and CYP3A4/3A5, followed by N-demethylation which produces 4-hydroxy-TAM, an active metabolite [15]. Analogs of TAM are being developed by researchers worldwide in a continuous manner with the goal of creating new treatment options for patients with BCs and other diseases, making TAM a significant drug template [15]. Currently, TAM remains an option in both early and advanced ER+ BCs in pre- and postmenopausal women [16,17]. TAM is also used for women at elevated risk of developing BC as a preventive solution. Studies showed that the ER expression level influences the tumor response to TAM and a fifteen-to-twenty-year benefit from TAM treatment in ER+ BCs [18,19]. However, it is important to note that studies with a follow-up extended beyond twenty years show fewer benefits of extended TAM administration as the annual recurrence rates were marked between fifteen and twenty-five years for BCs. This implies that for studies with a follow-up shorter than twenty-five years, the recurrence might not have been reached yet [20]. For two decades, studies have shown that TAM can be used either in the adjuvant setting in the first place or in sequence with other treatment options. The IMPACT (Immediate Preoperative Anastrozole, Tamoxifen, or Combined) study highlighted that AIs could provide more benefits than TAM if administered in the neoadjuvant setting in postmenopausal women diagnosed with ER+ BC. These results served for the establishment of the latest guidelines regarding TAM [13].

#### 2.2.2. Aromatase Inhibitors: AIs

Aromatase, the target of AIs, is responsible for the conversion of androgens to estrogens in peripheral tissues and plays a key role in ERα functions. In cases of BCs, abnormally elevated levels of aromatase can be associated with local production of estrogens. AIs were developed in response to these findings, with the goal of treating ER+ BC patients by blocking the synthesis of estrogens, reducing their levels by more than 90% in postmenopausal women [18,21]. They are sorted into three generations, depending on their efficiency in inhibiting aromatase. In addition to the three categories, they are further divided into two subtypes, depending on how reversible their action is. We will focus on the third generation of AIs (anastrozole (ANA), letrozole (LET), and exemestane (EXE)), as they represent the most efficient and least toxic AIs on the market [22]. ANA and LET are type I non-steroidal AIs, meaning they reversibly bind aromatase, whereas EXE is a type II steroidal AI, binding irreversible aromatase, and they are also known as “suicidal inhibitors” [4,23]. All three can be administered to postmenopausal women in early and advanced settings, but also to premenopausal women on the condition that they undergo ovary function suppression [4]. ANA was first introduced in the 1990s as an option for TAM failure in patients with early relapse or progression. ANA was also studied in a preventive setting for high-risk postmenopausal women, with a 61% reduction in BC incidence in the twelve-year follow-up [24]. In addition to ANA, LET has also proven to significantly improve time to progression compared to TAM in patients with ABC [25].

LET is a third generation of AIs and manages to achieve the highest level of estrogen suppression compared to other AIs. Due to its singular chemical structure, it offers the optimal fitting of the aromatase binding site out of the three aforementioned AIs [26]. Similarly, LET has shown efficacy in patients facing a TAM therapy failure and offers better efficacy in cases of ABC in postmenopausal women. Finally, the functions of EXE reside in its irreversible binding to the substrate-binding site. It offers a better overall response rate but no significant difference in overall survival (OS) compared to TAM [22,27]. The data of the SOFT-TEXT trials evaluate the effect of ovary function suppression (OFS) in combination with TAM or EXE in premenopausal women. The 12-year disease-free individuals showed an improvement of 4.6% in EXE + OFS versus TAM + OFS but no overall survival benefit [28].

#### 2.2.3. Selective Estrogen Receptor Downregulators: SERDs

The third category of ET globally available to treat BC is SERDs. They can be divided into two sub-categories depending on their chemical structure, whether they have an acrylic acid side chain or a basic amine side chain [29]. As of 2023, there are now two FDA-approved (Food and Drug Administration) SERDs on the market. Fulvestrant (FUL), the oldest marketed SERD, is derived from 17β-estradiol and binds competitively to ER, with an affinity higher than that of TAM. It functions in both the cytoplasm and the nucleus, either blocking the translocation of the receptor to the nucleus or the binding of coactivators. Contrary to TAM, FUL exerts an almost complete degradation of ER within the cell [30]. On its own, FUL can be administered to ER+ HER2− ABC patients alone or in combination with targeted therapies [31]. Two phase III trials (EFECT and SoFEA) showed an interest in *ESR1* mutation detection at baseline, as EXE versus FUL was associated with better progression-free survival (PFS) and OS in ER+ BC patients [32]. However, costs should be taken into consideration when deciding on treatment options, FUL being costlier than AIs and CDK4/6 inhibitors remaining the most cost-prohibitive solution [30,33].

Several phase III studies showed a lower PFS for AI and CDK4/6 inhibitor combinations [30]. A downside of FUL is its required intramuscular injection and poor bioavailability, which spurred further research into oral SERDs [13]. On 27 January 2023, a new oral SERD named Elacestrant (ELA) was approved by the FDA, supported by breakthrough results of the EMERALD Trial. ELA has a dose-dependent action on ERs, its chemical structure resembling that of TAM [34]. When tested on MCF-7 xenograft models, ELA proved to inhibit tumor growth to a greater degree than TAM and FUL. The phase I trial showed a drastic 89% reduction in estrogen receptors in tumors within two weeks [35].

The EMERALD study included both men and postmenopausal women with ABC presenting progression after one or two lines of ET and mandatory previous treatment of CDK4/6 inhibitors combined with FUL or AIs. ELA or standard of care (SOC) was randomly assigned (1:1) to patients [36], and the results highlighted a significant improvement in PFS for all patients treated with ELA compared to SOC. Results from patients with detectable *ESR1* mutations were included in this study, proving the advantage of ELA over SOC in ABC, with or without *ESR1* mutations [37].

Alongside ELA, several other novel SERDs are being developed to treat *ESR1*-mutated patients with HR+ BCs. Camizestrant (AZD9833) showed promising results, with a two-fold reduction in *ESR1* mutation levels in ctDNA of patients with a 14-day treatment in 92% of cases. Promising results in MCF-7 Y537S cells also suggest that Camizestrant can prevent proliferation in *ESR1* mutated cells [38]. The SERENA-1 trial evaluated the efficacy and dose-dependence of Camizestrant in ER+/HER2− BC patients and showed encouraging results for this molecule as a monotherapy [39]. The ongoing SERENA-6 trial aims to evaluate the interest in switching from AIs to Camizestrant in combination with CDK4/6 inhibitors in HR+/HER2− ABC patients as soon as an *ESR1* gene mutation is detected in ctDNA [40].

Giredestrant (GDC9545) is another orally available SERD currently tested for ER+/HER2− ABCs in males and post/premenopausal females with prior ET. Patients with detected *ESR1* mutations seem to reap more benefits from Giredestrant than FUL or AIs. Phase 3 trials are ongoing to evaluate its combination with CDK4/6 inhibitors and its efficiency compared to ET in the adjuvant setting [41].

Preclinical data showed promising results of Imlunestrant (LY-3484356) in *ESR1*-mutant models. The phase I EMBER study currently evaluates its efficacy in ER+/HER2− ABC patients in combination with CDK4/6 inhibitors with or without AIs [42].

Finally, the development of Amcenestrant has ceased following the negative results of the AMEERA-3 trial as it did not meet its primary objective. Indeed, in ER+/HER2− ABC patients, there was no improved PFS compared to the intention-to-treat population [43].

These findings highlighted the importance of detecting *ESR1* mutations when deciding on the more appropriate treatment course for ABC patients.

### 2.3. ESR1 Alterations: The Path to Resistance

**Figure 1:**
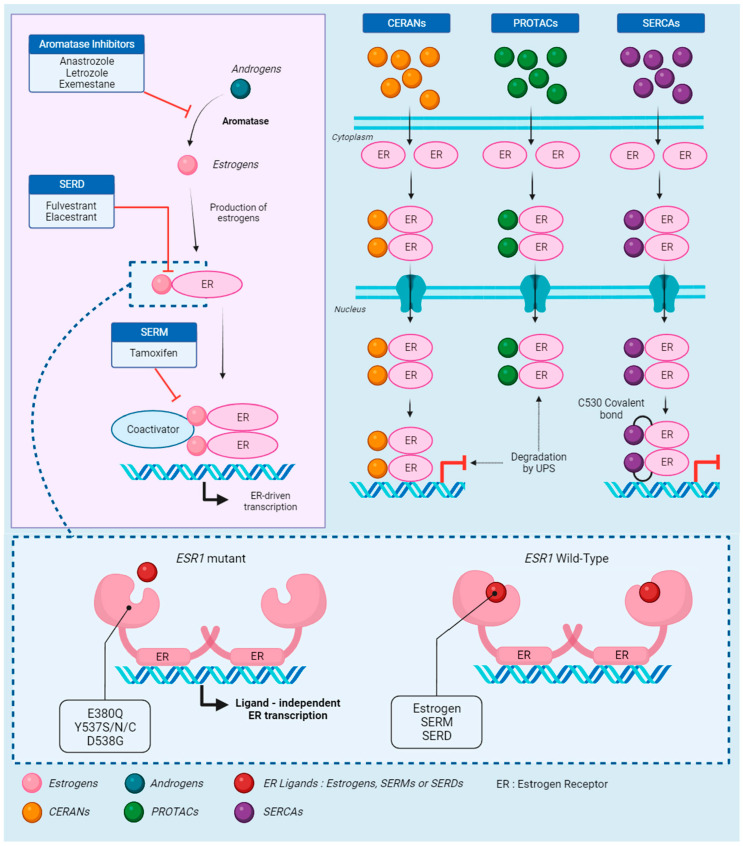
The estrogen receptor: signaling, treatment, and resistance. Androgens are the starting point of the estrogen receptor (ER) signaling pathway, quickly converted to estrogens by aromatase. This conversion is the target of aromatase inhibitors (AIs), which prevent the synthesis of estrogen. In absence of AIs, estrogens bind to the receptor, activating the recruitment of coactivators. Selective estrogen receptor downregulators (SERDs) block the binding of estrogen to the receptor, while selective estrogen receptor modulators (SERMs) block the coactivator recruitment. Those three endocrine therapies (ETs) effectively block the ER-driven transcription. However, in cases of mutations on the *ESR1* gene, which codes for ERs, estrogens, SERMs, and SERDs are unable to bind to the receptor. The activating mutations change the receptor to an apo conformation and allow ligand-independent transcription. Novel therapies were developed to respond to *ESR1*-driven ET resistance. Complete estrogen receptor antagonists (CERANs) bind to the receptor in place of estrogen and lead to its degradation by the ubiquitin–proteasome system (UPS) after DNA binding, while proteolysis targeting chimeras (PROTACs) induce degradation by UPS before DNA binding. Finally, selective estrogen receptor covalent antagonists (SERCAs) block the transcription by covalently binding to the specific *ESR1* residue C530.

Most ER+ ABC patients will develop ET resistance, either through the emergence of mutations in the receptor or activation of other involved pathways [44,45] (Figure 1). Additionally, some patients can suffer from intrinsic resistance to ET, meaning they never properly respond to treatment [46]. As stated previously, *ESR1* somatic mutations have been identified in the ET resistance associated with dysregulation of the receptor, leading to progression in ABCs. They are hardly detectable at diagnosis but can be found in up to 36% of ABCs previously treated with AIs and allow for constitutive activation of ERs in addition to decreased efficiency of ET [47,48].

**Figure 2:**
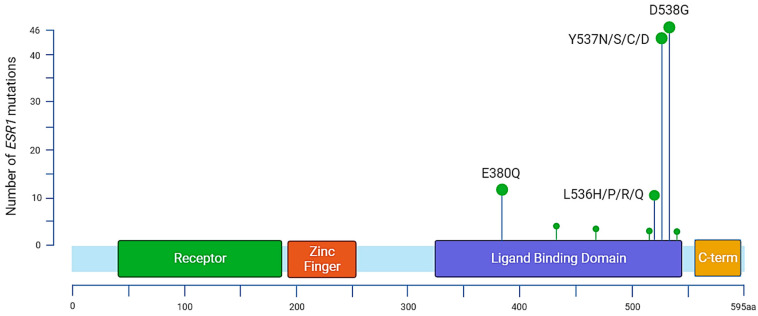
Distribution of annotated substitutions in the *ESR1* gene according to MSKCC-Impact data. The most prevalent mutations are located in the ligand binding domain. There are 13 identified mutations for variant E380Q, 3 substitutions for S432L, 3 for S463P, 1 for V534E, 12 for L536, 44 for Y537, 46 for D538, and 1 for L540.

In 1997, Zhang et al. characterized the first three *ESR1* mutations in metastatic BC samples, two of which were situated in the LBD. It constitutes the main region associated with ET resistance mutations and carries most of the hotspot mutations. Early BC patients [49] who completed at least 2 years of AI adjuvant treatment and experienced a documented relapse after the end of their treatment presented *ESR1* mutations in approximately 30% of cases, and an increase in mutation allele frequency (MAF) was associated with clinical deterioration. Other mutations have been reported throughout the *ESR1* gene, but around 90% of the characterized mutations associated with ET resistance remain Y537, D538, and E380 [50] (Figure 2, Table 1). Among the most prevalent point mutations of ERα, tyrosine on codon 537 (Y537) is the most frequently altered, as four different amino acid changes can be found in BC samples: Y537N, Y537S, Y537C, and Y357D. Other point mutations are also frequently found in ABCs, notably D538G and E380Q, and also affect the LBD [51]. The most frequent variant, Y537S, was found in 13.3% of patients included in the Bolero-2 trial (72 of 541), whereas the D538G variant was found in 21.1% of patients (114 of 541), giving a total of 28.8% patients with either of the mutations [52]. *ESR1* mutations impacting the LBD differentially affect the conformation of the estrogen receptor.

Y537 mutations create a switch in hydrogen bonds between Y537-N348 and Y537-D351, promoting the agonist conformation and provoking a ligand-independent activation of ERα [6,53]. The D538 substitution provides more flexibility to H12, resulting in better stability of the receptor and a heightened affinity with coactivators, also allowing a ligand-independent activation of ERα [54]. Interestingly, both Y537 and D538 point mutations work in the same fashion but do not result in the same level of resistance. Y537S binds to the Steroid Receptor Coactivator 3 (SRC-3) with more affinity than D538G in BC cell lines, which is probably responsible for the higher constitutive activity [6]. Moreover, studies on MCF-7 BC cell lines showed increased growth and higher transcriptional activation of ERs in cells harboring *ESR1* mutations [5].

In 1993, Pakdel et al. showed that the E380Q mutation allowed maximal activity of the receptor with a lower amount of estradiol required for activation than in wild-type (WT) *ESR1* [55]. Since then, this point mutation has been reported in ABCs, with prevalence ranging from 9.5% to 26% [56,57]. This point mutation also participates in ET resistance and increases sensitivity to estrogens and tumor growth; however, it does not seem to involve improved coactivator binding [55]. Li et al. also shed light on the potential role of *ESR1* mutations in corrupting the adhesive and migratory network, which can indirectly lead to ET resistance [48].

Since the number of detectable *ESR1* mutations rises throughout disease progression, this proves that *ESR1* mutations can be associated with an increased risk of ET resistance or ET resistance itself [58]. Martin et al. explained that FUL resistance resulting from *ESR1* mutations was due to the selection of clones presenting *ESR1* mutations at initial low frequencies by estrogen deprivation [59]. TAM can block the transcriptional activity of *ESR1* mutant cells, but the mutations reduce its binding capacity. Whether TAM can also block their growth remains to be proven [60]. Exposure to AIs is most commonly responsible for *ESR1* mutant clone selection, especially if administered in an advanced setting [61]. The SoFEA study highlighted that AIs (EXE) were associated with an almost four times shorter PFS if *ESR1* mutations were detected [56]. Finally, the resistance to FUL caused by *ESR1* mutations is questionable, notably because preclinical and clinical research show variable results. For example, preclinical work showed dose-related resistance to FUL [62], and the PALOMA-3 trial showed a positive selection of Y537 and potentially shorter PFS [63].

Recently, *ESR1* fusions have been reported in ER+ ABC, but information regarding their clinical effect is lacking. After analyzing tissue and circulating tumor DNA samples, results showed five *ESR1*-specific fusions disrupting the LBD, thus resulting in the ligand-independent activity of the receptor [64,65]. However, it is important to note that LBD loss caused by *ESR1* fusions may not be detected by liquid biopsy-based techniques, implying that this source of resistance remains to be further elucidated [66].

### 2.4. Novel Strategies to Overcome ESR1-Mediated Resistance

With the rise of new oral SERDs and *ESR1* resistance, several novel strategies are being developed to overcome these challenges. Proteolysis targeting chimeras (PROTACs) degrade specific proteins through the E3 ubiquitin ligase pathway [67]. Clinical trials showed that the PROTAC molecule ARV-471 could significantly reduce ER levels by over 60% in patients suffering from ER+ HER2− BCs [68]. A patient with an identified D538G mutation and extensive prior therapies showed a confirmed partial response and targeted lesion reduction with ARV-471 [69]. AC682 is also a promising PROTAC drug for patients with known Y537S and D538G *ESR1* mutations, with more than 90% tumor reduction and ER expression reduction in animal tumor models [70].

With the same mindset, selective estrogen receptor covalent antagonists (SERCAs) were developed to target mutant ERα. H3B-5942 inactivates both *ESR1* Y537S mutant and WT receptors by targeting Cysteine 530 (C530), a residue specific to ERα [71]. More recently, H3B-6545 was designed to overcome liabilities in H3B-5942, as it was highly dependent on the engagement with C530. A clinical study based on encouraging preclinical results on patient-derived xenograft (PDX) models harboring Y537S mutations is ongoing to assess the potential of H3B-6545 in patients with ER+ HER2− ABCs [72].

Finally, a third type of novel therapy was recently developed: complete estrogen receptor antagonists (CERANs). Their action relies on the inhibition of both transcription activation sites AF1 and AF2 of ERα. A phase I/II trial is evaluating the molecule OP-1250 in HR+ ABC cases in post and premenopausal women based on preclinical studies that showed promising results in WT and Y537S mutant ERs [73,74]. Studies involving new oral SERDs and other therapies are listed in Table 2. All these new therapies are a positive outcome of the ongoing search for new treatment options in ER mutant ABCs, bringing hope to patients suffering from ET resistance (Figure 1).

## 3. Techniques for Identification of *ESR1* Mutations

Over the years, several methods have been developed with the goal of studying the genomic profile of tumors. Tumors release ctDNA into the bloodstream, allowing for the detection of mutation tumor alterations through the analysis of plasma samples called “liquid biopsy”. Since normal cells can also release their genomic material in body fluids, ctDNA represents a limited portion of the total cell-free DNA (cfDNA), thus requiring ultra-sensitive strategies to be able to detect tumor alterations. ctDNA/cfDNA ratio strongly depends on tumor stage, histological type, and tissue of origin, making the analysis of ctDNA challenging in many cases [75,76].

### 3.1. The Concept of Liquid Biopsy: A Small Step Forward Personalization of Treatment

In oncology, the identification of tumor-specific gene alterations has become essential for tumor stratification and treatment guidance, thus providing each patient with a personalized therapeutic approach. In most cases, a tumor molecular diagnosis is performed using DNA extracted from a biopsy or surgical specimen of a primary or metastatic site. However, a tissue biopsy only provides information limited to the sampling site without being able to inform on the heterogeneity existing within a bulky tumor or even between several metastatic sites. Multiple biopsies can help to better decipher tumor heterogeneity but increase the risk of associated morbidity. Consequently, resampling of tissue biopsy over the disease course seems not to be adapted in clinical practice to follow the dynamic evolution of the tumor under the influence of micro-environmental stimuli and clonal selection due to therapeutic pressure.

These points justify the development of alternative strategies that can provide the same information as conventional biopsy techniques but in a less invasive way for the patient. During tumor growth, tumor cells release their content, including DNA, into the bloodstream, and therefore, the plasma contains a representation of the tumor genome. The molecular diagnosis of cancers based on the analysis of ctDNA, which is called “liquid biopsy”, has progressed during the last decades with the emergence of techniques capable of identifying very low mutant allele frequencies (MAFs) [77]. The quantity of circulating tumor DNA represents only a minor fraction of the total circulating free DNA, frequently less than 1%.

### 3.2. Next Generation Sequencing: NGS

Circulating tumor DNA extracted from liquid biopsies can be analyzed using Next Generation Sequencing (NGS). This strategy represents a considerable advantage for the implementation of personalized medicine. In order to identify *ESR1* mutations in blood samples from ABC patients, NGS can be the preferred technique and shows the possibility of detecting non-hotspot and non-reported mutations [78,79,80]. It represents about 50% of the molecular assays used for *ESR1* mutation detection in current practice. Scientists can choose from three distinct strategies in terms of NGS sequencing. The first is Targeted Panel Sequencing, which is a more focused approach and allows for the sequencing of a selected panel of genes with a high diagnostic yield [81]. Guardant360, a commercially available targeted sequencing approach, is one of the first FDA-approved techniques to combine NGS and liquid biopsy. It allows practicians to obtain the analysis of 73 genes within seven days and guides earlier treatment decisions [82]. Guardant360 CDx, an updated version of Guardant360, was developed for ctDNA analysis and serves as the companion test associated with the newly FDA-approved Elacestrant [83].

The MSKCC-IMPACT is also a great example of targeted sequencing, used to study the link between *ESR1* mutations and ET efficiency [79]. Whole Exome Sequencing (WES) strategies target around 22,000 protein-coding genes and provide a great compromise between costs, coverage, and diagnostic yield. This method requires high-throughput in silico analysis in order to interpret raw data for clinical purposes. However, even though WES covers the entire exome, it only targets 1–2% of the complete genome [84]. Finally, the third strategy, the most extensive and expensive one, is Whole Genome Sequencing (WGS). It has already been used to study *ESR1* mutations in cases of ET resistance [85]. Additionally, most NGS strategies allow the detection of other alterations related to ET resistance, such as mutations affecting the PI3K/AKT/mTOR pathway or genes involved in the regulation of the cell cycle.

**Figure 3:**
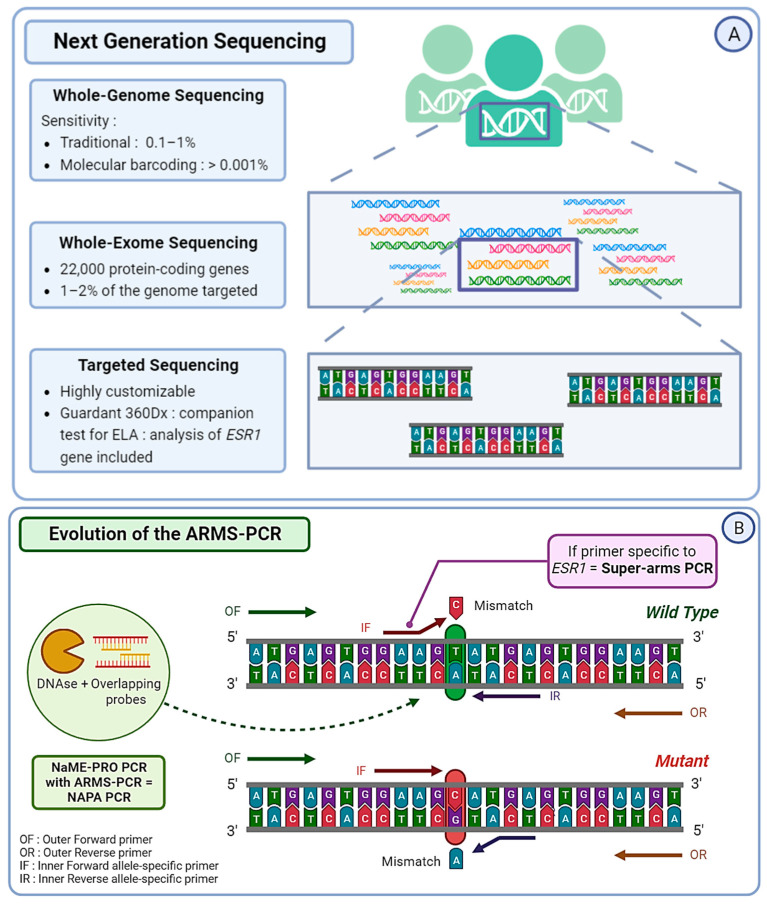

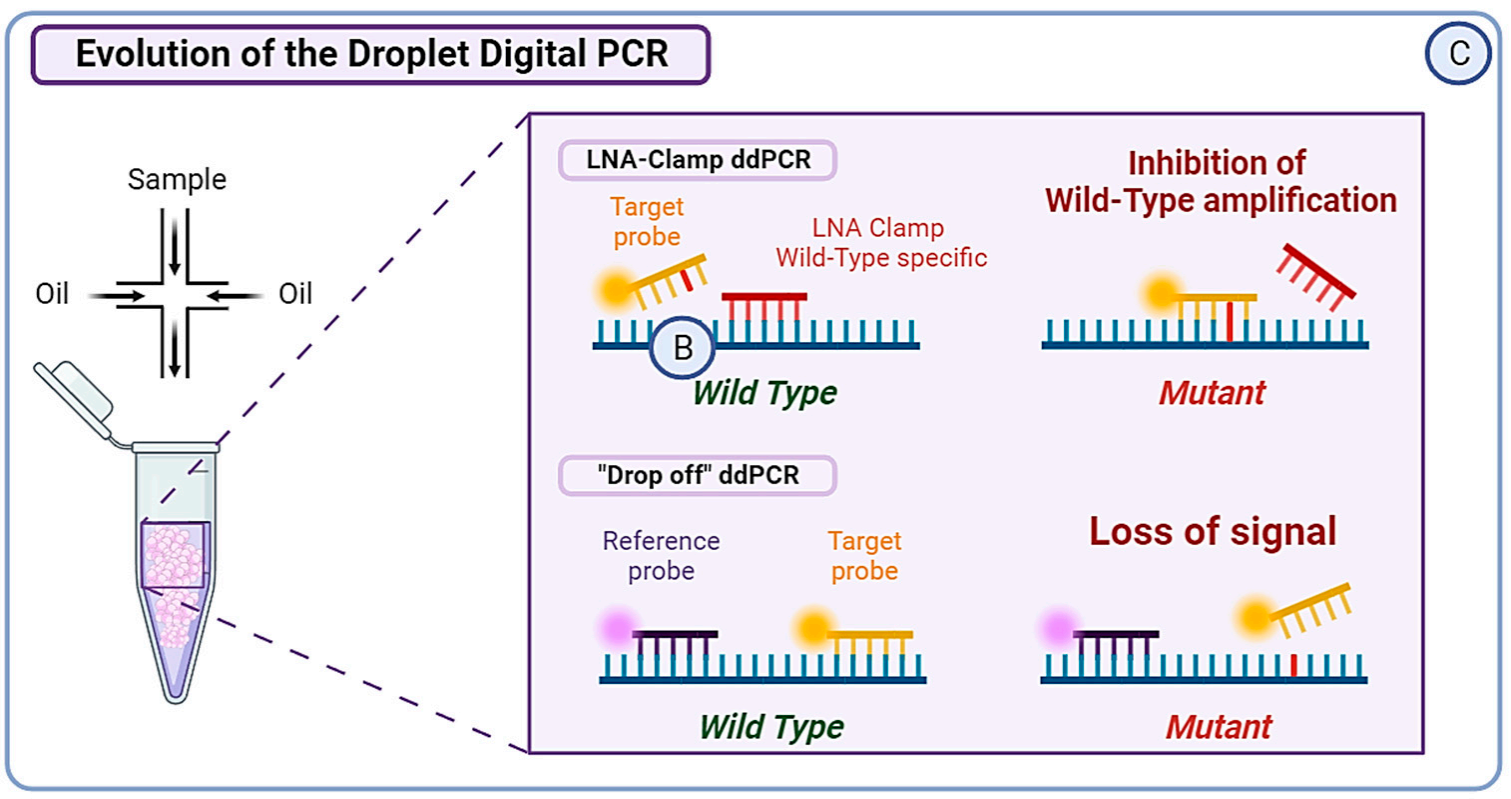
(**A**) Next Generation Sequencing (NGS) can be divided into three categories. Whole Genome Sequencing (WGS) covers the entire genome and has a limit of detection of approximately 0.1–1%, albeit it can reach 0.001% if molecular barcoding is used. Whole Exome Sequencing (WES) analyzes around 22,000 protein-coding genes, which represent only 1–2% of the whole genome. Targeted sequencing, which is a highly customizable approach, covers only specific regions of the genome or panels of genes. For example, Guardant360CDx is a commercial gene panel including the *ESR1* gene, used as a companion test to guide ELA treatment. (**B**) The Amplification Refractory Mutation System (ARMS) PCR is a technique requiring four primers, with two outer gene-specific primers and two inner allele-specific primers. Here, the inner forward primer is specific to the mutant allele, whereas the inner reverse primer is specific to the wild-type (WT) allele. This will allow the outer primers to amplify the sequence, but also either of the inner–outer couples of primers. This produces three types of amplicons: one wild-type and one of each extremity of the mutant allele. In order to increase the specificity of this method, the Super-arms technique was designed with inner primers specific to *ESR1* gene mutations. Finally, to reduce the number of WT amplicons produced by the approach, a DNA-specific nuclease (DNAse) can be used for Nuclease-assisted Minor Allele Enrichment using probe overlap (NAME-PRO) PCR. The combination of this method with the ARMS-PCR (NAPA-PCR) allows for the inhibition of WT amplification as the DNAse recognizes and degrades the WT DNA hybridized with the overlapping probes. (**C**) Two methods derived from conventional droplet digital PCR (ddPCR) approach were designed to improve its sensitivity and specificity. The locked nucleic acid (LNA) clamp ddPCR uses a wild-type (WT) specific probe that hybridizes with the WT DNA, preventing the fixation of target probe and thus blocking the fluorescent signal. Only the mutant sequence will allow the hybridization of the target probe and produce a signal. Another approach called the “drop-off” PCR was employed in several studies, including the PADA-1 trial. Here, two probes are used with different fluorophores. The reference probe is hybridized with a constant sequence and is combined with a target probe that is specific to the WT DNA sequence of interest. When encountering a mutated sequence, the target probe will not hybridize, resulting in only one of the two signals. With this technique, the difference in fluorescence is used to measure the number of mutated amplicons.

The use of molecular barcodes (MBs) in NGS can provide a detection sensitivity level as low as 0.001%, whereas the sensitivity level of traditional NGS without MBs is around 0.1–1% [86]. The limitation of these techniques lies in the amount of ctDNA available for analysis, often lower than ideal, which is why existing techniques can be adapted to fit practical limitations (Figure 3A). Masunaga et al. based their MB-NGS methods on the Safe-seq sequencing solution specifically for *ESR1* mutation detection. This method relies on the use of unique molecular identifiers (UMIs) in order to reduce PCR artifacts and reliably detect rare variants [87]. They managed to detect mutations with a mutation allele frequency (MAF) as low as 0.1% and a lower error rate (<0.625%) than standard NGS [88]. NGS can also be used as a cross-validation technique in cases of validation of the method, a great example being the cross-validation of droplet digital PCR (ddPCR) for the real-time detection of *ESR1* mutation in the PADA-1 trial (PAlbociclib and Circulating Tumor DNA for ESR1 Mutation Detection) [89].

### 3.3. Polymerase Chain Reaction: PCR

Next Generation Sequencing is an effective method, but it often comes with a high cost and lengthy procedures. To overcome those limitations, several optimizations of the traditional PCR were developed. The Amplification Refractory Mutation System (ARMS) PCR relies on sequence-specific PCR primers that target and amplify solely the allele present within the sample, followed by a standard PCR procedure [90]. However, the number of false positives due to WT DNA makes the conventional ARMS-PCR protocol not suitable for *ESR1* mutation detection in blood. Chen et al. conceived enhanced primers specific to *ESR1* and managed to obtain a detection level close to that of ddPCR, with a smaller cost and a faster technique. They named it “Super-arms” [91]. In the same context, Stergiopoulou et al. combined another process of Nuclease-assisted Minor Allele Enrichment using Probe Overlap (NaME-PrO) with the ARMS to obtain a NAPA (NaME-PrO-assisted ARMS) PCR. The goal was to remove most of the WT DNA molecules and create a more reliable detection tool for *ESR1* mutations in blood samples. With this approach, they reached a 90.6% concordance with ddPCR, with a reduced cost and time [92] (Figure 3B). More recently, Kojima et al. optimized a peptide nucleic acid (PNA)-based PCR to increase the sensitivity of detection of polyclonal *ESR1* mutations in ctDNA, creating a PNA-locked nucleic acid (LNA) mediated PCR clamp (PNA-LNA PCR clamp). The results are promising and require further validation in order to be utilized in a clinical setting [93]. More PCR-based techniques are being optimized by scientists worldwide and show their will to create ultra-sensitive alternative options to optimally detect *ESR1* mutations in blood samples.

### 3.4. Droplet Digital PCR: ddPCR

In recent years, numerous studies have evaluated the use of ddPCR to identify *ESR1* mutations, with a theoretical sensitivity reported as low as 0.001%. However, the presence of droplets above the threshold of detection reduces the sensitivity to a more realistic 0.01% [94]. Now, ddPCR is the most commonly used digital PCR method for the analysis of ctDNA. The major advantage of ddPCR over PCR lies in its calibration-free quantification [95]. In the PADA-1 trial, a ddPCR system was used to target exons 5 and 8 of the *ESR1* gene. The mutation allele frequency (MAF) of the mutations detected with ddPCR greatly matched the cross-validation MAF from NGS (intraclass correlation coefficient of 0.93; 95% CI [0.85; 0.97]) [89]. In order to detect rare *ESR1* mutations, Hashimoto et al. combined the aforementioned LNA-clamp method with ddPCR, allowing the inhibition of WT DNA amplification. This technique proved useful in detecting *ESR1* variants with a VAF < 0.1% in primary BC tumors, which would have been missed with conventional techniques [96]. Another research group developed a multiplex ddPCR combined with “drop-off” probes. The “drop-off” probes lead to a loss of signal if a single mismatch occurs compared to WT samples. They succeeded in detecting up to eight different hotspot ESR1 mutations in a single reaction with a limit of detection ranging from 0.07 to 0.19% in MAF. Moreover, the approach permitted the detection of polyclonal *ESR1* mutations frequently observed in patients, making it suitable for real-time follow-up liquid biopsy [97] (Figure 3C).

## 4. Liquid Biopsy for Monitoring *ESR1* Mutations and Personalization of Endocrine Therapy

As mentioned previously, techniques for the detection and analysis of ctDNA can be divided into targeted techniques and non-targeted techniques able to screen many genes, from several tens or hundreds to the entire genome. The concept of liquid biopsy thus finds its applications from screening to therapeutic monitoring of cancers. For example, the use of liquid biopsy has now been implemented for several years for therapeutic purposes and the detection of resistance mechanisms in non-small cell lung cancer patients with *EGFR* mutations treated with anti-EGFR therapies [98]. More recently, using liquid biopsy was extended to all situations in which molecular biology analyses could not be carried out from a tissue sample.

Additionally, liquid biopsy provides insight into tumor burden through follow-up analysis of ctDNA. Indeed, longitudinal detection of mutations can portray tumor heterogeneity and clonal evolution and allow for early assessment of disease progression [99]. Follow-up using liquid biopsy is now used in more clinical trials [100] and could benefit patients in a routine setting.

### Clinical Utility of the Detection of ESR1 Mutation in Liquid Biopsies

The association between *ESR1* gene mutations and ligand-independent activation of ER, as well as resistance to TAM and FUL, has been known for a decade [101,102,103]. However, their clinical significance was not considered since their presence in primary disease remains rare, but they are progressively being taken into account. The metastatic location, the age of the patient, or the presence of HER2 amplifications do not seem to influence the frequency of these mutations [52]. It was first reported that the detection of *ESR1* mutations in ctDNA in 39.1% of metastatic patients appears to correlate with clinical resistance to AIs [104]. This study also showed a strong concordance between the type of *ESR1* mutation found in tissue biopsies and plasma samples for seven advanced HR+ patients treated with anti-aromatases. Therefore, monitoring *ESR1* gene mutation in ABC progressing on AIs using liquid biopsy [105] showed that *ESR1* mutations were detected before the occurrence of any clinical or radiological tumor progression in up to 75% of cases [106].

The detection of *ESR1* resistance-associated mutations in primary disease is also relevant, even considering the low rate of mutations at diagnosis. Recently, an analysis of 3217 primary BC samples showed that 0.9% of patients had detectable *ESR1* mutations. This highlights the importance of early and continuous screening of *ESR1* mutations in order to properly orientate treatment, for example, preferring FUL administration to mutated patients [107].

In the BOLERO-2 study [52], approximately 30% of the patients treated with AI developed *ESR1* mutation and had reduced OS, 25.99 months for D538G [95% CI, 19.19–32.36 months], 19.98 months for Y538S [13.01–29.31 months] and an even lower OS (15.15 months [95% CI, 10.87–27.43 months]) if the patient harbors both mutations [52]. The addition of Everolimus (EVE, an mTOR inhibitor) to AIs led to an increase in PFS in either WT or mutant cases. In the SoFEA trial, circulating *ESR1* mutation was detected in 39% of cases, 49.1% of which were polyclonal. For patients with *ESR1* mutations, the PFS was improved after taking FUL compared with EXE (hazard ratio [HR], 0.52; 95% CI, 0.30 to 0.92; *p* = 0.02), whereas patients with WT *ESR1* had similar PFS with FUL and EXE (HR, 1.07; 95% CI, 0.68 to 1.67; *p* = 0.77) [56]. In the PALOMA-3 trial, *ESR1* mutations were found in the plasma of 25% of patients, harboring mutations associated with acquired resistance to prior AIs. FUL plus Palbociclib (PAL) improved PFS compared with FUL plus placebo in both *ESR1* mutant (HR, 0.43; 95% CI, 0.25 to 0.74; *p* = 0.002) and *ESR1* wild-type patients (HR, 0.49; 95% CI, 0.35 to 0.70; *p* = 0.001) [56]. In both the SoFEA and PALOMA-3 trials, there was no significant difference between the specific *ESR1* mutations and WT *ESR1* when assessing the effect of treatments on PFS [56].

Taken together, the results gathered from the retrospective or prospective–retrospective analysis of archived plasma specimens highlighted the usefulness of liquid biopsy for monitoring the occurrence of *ESR1* mutation in patients with ABC treated by ET (Table 2). Based on these findings, prospective trials were initiated, notably PADA-1 [108] and CICLADES trials [109]. The results of the PADA-1 trial were recently reported and show the feasibility of large-scale, real-time serial monitoring and targeting of resistance-associated mutations by ctDNA analysis. The PADA-1 study showed that, in the case of *ESR1* mutation detection in ctDNA, switching of ET improved PFS. These results confirm the clinical utility of monitoring ctDNA to detect *ESR1* mutations as an early biomarker of ET resistance in ABC [110].

Contrary to the PADA-1 trial, the phase II MAINTAIN trial randomly tested Ribociclib (CDK4/6 inhibitor) versus placebo, associated with a switched ET in progressing ABC patients. The PFS was improved for patients treated with a new ET plus Ribociclib, with a median PFS of 5.29 months versus 2.76 months for patients treated with a placebo. In this study, the presence of *ESR1* gene mutations at baseline led to similar results in FUL plus Ribociclib versus FUL plus placebo. However, the cohort studied was small and should require further investigation regarding the reasons for these results [111].

Acknowledging the clinical utility of detecting *ESR1* mutation in BC, clinical strategies taking into consideration *ESR1* mutations in HR+ HER2− ABC were evaluated. Within these strategies, innovative oral SERDs, such as ELA, were evaluated. In the EMERALD trial [36], the patients were randomly assigned to ELA versus SOC endocrine monotherapy. The primary endpoints were PFS in all patients and patients with detectable *ESR1* mutations. In this trial, *ESR1* mutation was detected in 48% of patients; PFS was prolonged in all patients (hazard ratio = 0.70; 95% CI, 0.55 to 0.88; *p* = 0.002) and in patients with *ESR1* mutation (hazard ratio = 0.55; 95% CI, 0.39 to 0.77; *p* = 0.0005). This pivotal trial led to the approval by the FDA of ELA for postmenopausal women or adult men with ER-positive, HER2-negative, and *ESR1*-mutated ABC with disease progression following at least one line of endocrine therapy.

In such a context, ASCO Rapid Recommendations Updates were recently published [2] to test for *ESR1* mutations to guide therapy for HR+ HER2− ABC. Routine testing is recommended for the detection of *ESR1* mutations at recurrence or progression on ET (associated or not with CDK4/6 inhibitor) in patients with ER-positive HER2− ABC. Testing should be performed on blood or tissue obtained at the time of progression, as *ESR1* mutations rise in response to selection pressure during treatment and are typically undetectable in the primary tumor. Blood-based ctDNA is preferred owing to greater sensitivity. Patients whose tumor or ctDNA tests remain *ESR1* wild-type may warrant retesting at subsequent progression to monitor the rise of *ESR1* mutations. Patients previously treated with ET and a CDK4/6 inhibitor for ABC have several therapeutic options. For patients with prior CDK4/6 inhibitor treatment and *ESR1* wild-type tumors, appropriate subsequent ET options include FUL, aromatase inhibitor, or TAM monotherapy. A combination of ET and targeted agents, such as alpelisib for PIK3CA-mutated tumors or EVE, is also an option. For patients with prior CDK4/6 inhibitor treatment and a detectable *ESR1* mutation, options include ELA or the same combinations offered to wild-type *ESR1* patients. There is no clinical evidence to support the use of ELA in combination with targeted agents.

## 5. Conclusions

### A Giant Leap for Personalization of Endocrine Therapy in Breast Cancer?

*ESR1* mutations remain extremely rare at the initial diagnosis of BCs and only occur at detectable levels once the disease progresses. However, since they are the main somatic resistance mutations acquired during ET, their early detection based on the analysis of serial liquid biopsy samples should be strongly considered in routine practice, especially at progression after ET, with the aim of treatment personalization in ABC. In order to detect ESR1 mutations more accurately and predict progression, the time of testing can be determined according to the PFS data observed in clinical trials such as PADA-1. It is also important to remember that novel therapeutic solutions still provide modest benefits, and their impact on survival remains to be confirmed with larger studies in both early and advanced breast cancer.

Overall, this innovative procedure of follow-up liquid biopsy to monitor *ESR1* mutation can represent a giant leap for the personalization of endocrine therapy in breast cancer.

## Figures and Tables

**Table 1:** Summary of activating *ESR1* substitutions *(NM_000125.4)*.

Variant	Exon and Domain	CDS Mutation	AA Mutation	dbSNP Reference	Clinical Impact
E380Q	Exon 5—LBD	c.1138G>C	p.(Glu380Gln)	rs1057519827	Likely pathogenic
S463P	Exon 7—LBD	c.1387T>C	p.(Ser463Pro)	rs1057519714	Pathogenic
L469V	Exon 7—LBD	c.1405C>G	p.(Leu469Val)	rs2050452121	GOF
L536H	Exon 8—LBD	c.1607T>A	p.(Leu536His)	/	GOF
L536P	Exon 8—LBD	c.1607T>C	p.(Leu536Pro)	/	GOF
L536R	Exon 8—LBD	c.1607T>G	p.(Leu536Arg)	rs1057519717	Pathogenic
Y537N	Exon 8—LBD	c.1609T>A	p.(Tyr537Asn)	/	GOF
Y537S	Exon 8—LBD	c.1610A>C	p.(Tyr537Ser)	/	GOF
Y537C	Exon 8—LBD	c.1610A>G	p.(Tyr537Cys)	/	GOF
Y537D	Exon 8—LBD	c.1609T>G	p.(Tyr537Asp)	/	Likely GOF
D538G	Exon 8—LBD	c.1613A>G	p.(Asp538Gly)	/	GOF
L540Q	Exon 8—LBD	c.1619T>A	p.(Leu540Gln)	/	Inconclusive

Ligand binding domain (LBD), CoDing Sequence (CDS), amino acid (AA), Single Nucleotide Polymorphism Database (dbSNP), and Gain Of Function (GOF).

**Table 2:** Summary of ongoing and completed trials evaluating therapies in ABC.

Treatment		Randomization	Study NCT Registry Number	Phase and Status	Population	**Main Results**
Main Molecule	Combination					
SOF	TAM or EXE	Yes vs. TAM alone	SOFT NCT00066690	III Active, not recruiting	Premenopausal HR+ BC	In premenopausal women, EXE + SOF has more efficacy than TAM alone and TAM + SOF
EXE	Triptorelin	Yes vs. TAM + Triptorelin	TEXT NCT00066703			
FUL	/	Yes vs. EXE	EFECT NCT00065325	III Completed	HR+ ABC	FUL and EXE are equally efficient in patients with progression on or recurrence of AIs
FUL	ANA (optional)	Yes vs. EXE	SOFEA NCT00253422	III Completed	ER+ locally ABC	If resistance to AIs arises, FUL + ANA shows no better efficacy than FUL alone or EXE
ELA	/	Yes vs. SOC	EMERALD NCT03778931	III Active, not recruiting	ER+/HER2− ABC	ELA provides a better PFS than SOC in *ESR1* mutated patients
Camizestrant	Multiple combination	No	SERENA-1 NCT03616587	I Recruiting	ER+/HER2− ET resistant ABC	/
Camizestrant	CDK4/6 inhibitors	Yes vs. AI	SERENA-6 NCT04964934	III Recruiting	HR+/HER2− Metastatic BC	/
Giredestrant	PAL	Yes vs. LET + PAL	persevERA NCT04546009	III Active, not recruiting	ER+/HER2− locally ABC	/
Imlunestrant	Monotherapy	Yes vs. anticancer therapies	EMBER NCT04188548	Ia/Ib Active, not recruiting	ER+ locally ABC and selected non-BC	Efficacy in ER+/HER2− ABC patients with *ESR1* mutations and resistance to CDK4/6 inhibitors and FUL
Imlunestrant	Abemaciclib	Yes vs. investigator choice	EMBER-3 NCT04975308	III Recruiting	ER+/HER2− ABC	/
Amcenestrant	/	Yes vs. physician choice	AMEERA-3 NCT04059484	II Active, not recruiting	ER+/HER2− ABC	Primary objectives of improved PFS not achieved
ARV-471	PAL (optional)	No	NCT04072952	I/II Recruiting	ER+/HER2− locally ABC	/
AC682	/	No	NCT05080842	I Recruiting	ER+/HER2− locally ABC	/
H3B-6545	/	No	NCT03250676	I/II Active, not recruiting	ER+/HER2− locally ABC	/
OP-1250	PAL	No	NCT05266105	I Recruiting	ER+/HER2− ABC	/
PAL	AI	No	CICLADES NCT03318263	/ Completed	ER+/HER2− Metastatic BC	/
PAL	AI	Yes vs. FUL	PADA-1 NCT03079011	III Active, not recruiting	ER+/HER2− Metastatic BC	Combination of FUL + PAL in *ESR1* mutated patients is more efficient than AI + PAL
EXE	EVE	Yes vs. placebo	BOLERO-2 NCT00863655	III Completed	ER+/HER2− locally ABC	Combination of EXE + EVE over EXE alone increases PFS but not OS
FUL	PAL (optional)	Yes vs. placebo	PALOMA-3 NCT01942135	III Completed	HR+/HER2− Metastatic BC	In ET-sensitive patients, FUL + PAL improves PFS; OS is not improved in the entire trial group
FUL	/	Yes vs. Ribociclib	MAINTAIN NCT02632045	II Active, not recruiting	HR+/HER2− ABC	/

Orange: Novel oral SERD studies; yellow: PROTAC study; blue: SERCA studies; and green: CERAN study.
